# Climate Change, Human Health, and Biomedical Research: Analysis of the National Institutes of Health Research Portfolio

**DOI:** 10.1289/ehp.1104518

**Published:** 2013-01-18

**Authors:** Christine M. Jessup, John M. Balbus, Carole Christian, Ehsanul Haque, Sally E. Howe, Sheila A. Newton, Britt C. Reid, Luci Roberts, Erin Wilhelm, Joshua P. Rosenthal

**Affiliations:** 1 Fogarty International Center,; 2 National Institute of Environmental Health Sciences,; 3 Division of Program Coordination, Planning, and Strategic Initiatives,; 4 National Library of Medicine,; 5 National Cancer Institute, and; 6 Office of Extramural Research, National Institutes of Health, Department of Health and Human Services, Bethesda, Maryland, USA

**Keywords:** climate change, climate variability, health impacts, health research, research portfolio

## Abstract

Background: According to a wide variety of analyses and projections, the potential effects of global climate change on human health are large and diverse. The U.S. National Institutes of Health (NIH), through its basic, clinical, and population research portfolio of grants, has been increasing efforts to understand how the complex interrelationships among humans, ecosystems, climate, climate variability, and climate change affect domestic and global health.

Objectives: In this commentary we present a systematic review and categorization of the fiscal year (FY) 2008 NIH climate and health research portfolio.

Methods: A list of candidate climate and health projects funded from FY 2008 budget appropriations were identified and characterized based on their relevance to climate change and health and based on climate pathway, health impact, study type, and objective.

Results: This analysis identified seven FY 2008 projects focused on climate change, 85 climate-related projects, and 706 projects that focused on disease areas associated with climate change but did not study those associations. Of the nearly 53,000 awards that NIH made in 2008, approximately 0.17% focused on or were related to climate.

Conclusions: Given the nature and scale of the potential effects of climate change on human health and the degree of uncertainty that we have about these effects, we think that it is helpful for the NIH to engage in open discussions with science and policy communities about government-wide needs and opportunities in climate and health, and about how NIH’s strengths in human health research can contribute to understanding the health implications of global climate change. This internal review has been used to inform more recent initiatives by the NIH in climate and health.

Global climate change is anticipated to have multiple impacts on human health, many of them adverse and some severe, but most of these impacts are poorly understood [Climate Change Science Program (CCSP) 2008; Intergovernmental Panel on Climate Change (IPCC) 2007]. The National Institutes of Health (NIH) has been urged by members of the research and advocacy communities (e.g., Ebi et al. 2009), by the World Health Organization (e.g., Chan 2007), and by experts within the U.S. government (Portier et al. 2010) to address the issue. The NIH has taken a strategic approach, including the formation of an agency-wide Working Group on Climate and Health (Glass et al. 2009), to conduct a gaps analysis and help develop a research agenda. One of the first steps was a portfolio analysis to better understand the gaps in current and historical activities in relation to the types of climate and health research urged by these stakeholders. The NIH has since begun to address those gaps with targeted funding opportunities. In this commentary we present the results of a comprehensive project funding analysis and briefly describe some of the funding efforts that have followed.

The pathways by which climate change is projected to affect human health range from relatively straightforward effects on heat stress and heat mortality (Luber and McGeehin 2008; McGeehin and Mirabelli 2001) to more complex effects on infectious and other diseases (Lafferty 2009; Paaijmans et al. 2009). Other secondary and more complex pathways may include population migration or human conflict arising from food or water scarcity brought on or exacerbated by climate change. These varied potential impacts are likely to be more severe for populations and geographic regions already experiencing a high burden of public health problems and resource scarcity (Tang et al. 2009).

As manifestations of global climate change become more apparent, the scientific community is placing increasing emphasis on research and science-based decision making for responding to climate change (CCSP 2008; IPCC 2007; Semenza and Menne 2009). In addition, actions to reduce greenhouse gas emissions (including carbon dioxide, which is produced by burning fossil fuels) or reduce vulnerability to climate change impacts may themselves have both positive and negative health consequences that need to be assessed in decision making (Haines et al. 2009). Because of the inherent uncertainties in predicting changes in Earth’s climate and especially in the subsequent chain of effects on ecosystems, human systems, and human health, research is needed to improve scientific understanding of these complex interactions and to improve the ability to identify, prevent, and respond to the more serious potential health consequences (Rosenthal and Jessup 2009).

The report titled *A Human Health Perspective on Climate Change: A Report Outlining the Research Needs of the Human Health Effects of Climate Change*, by the ad hoc Interagency Working Group on Climate Change and Health, outlines research needs for climate change and human health (Portier et al. 2010). The report, organized around 11 categories of health outcomes, describes the linkages between climate change and disease-specific federal health research priorities. The ad hoc group has been replaced by a chartered Interagency Crosscutting Group on Climate Change and Human Health (CCHHG) under the U.S. Global Change Research Program (USGCRP 2012). As the CCHHG plans, coordinates, implements, evaluates, and reports on research related to the human health impacts of global environmental change, it seeks to foster interagency, interdisciplinary, and intergovernmental collaborations (CCHHG 2012).

Among the agencies represented in the CCHHG, the NIH is unique in its mission to seek fundamental knowledge about the nature and behavior of living systems and the application of that knowledge to enhance health, lengthen life, and reduce the burdens of illness and disability. Approximately 80% of the $30.9 billion fiscal year (FY) 2011 budget (NIH 2012c) of its 27 components [called institutes and centers (ICs)] supports research and research training across a wide variety of disciplines and health outcomes through nearly 53,000 grants and contracts. The overwhelming majority of this research support is awarded through peer review of individual grant applications submitted by scientists. Approximately 90% of the funded applications are unsolicited investigator-initiated efforts (NIH 2012a). The remainder are responses to solicitations [e.g., requests for applications (RFAs) and program announcements (PAs)] for grant applications to address defined research topics or enhance particular program areas. Projects range from genome sciences and basic biology to drug and vaccine clinical trials, community- and population-based research, and the development of information resources to enhance health and research, among others. Although most NIH-supported research addresses basic human biology and the development of interventions for the prevention and treatment of disease, some address policy development. Examples include the effects of tobacco taxes and prices on tobacco consumption and associated diseases, and the use of dose–response curves of human and animal exposures to industrial pollutants to inform regulations.

To develop a more comprehensive research agenda on climate change and human health, it was first necessary to identify and analyze the current NIH research portfolio relevant to climate change, so that research gaps could be identified and prioritized. By sharing these findings widely, we hope to generate further input for this evolving research agenda.

## Methods

The goals of this analysis were to identify the projects related to climate and health, assess the degree to which each project focused on the intersection on climate and health, and characterize each climate and health project according to one or more of four criteria: climate pathway, health impact, study type, and study objective. The analysis was a multistage effort. The input data for this analysis consisted of the list of almost 53,000 awards that the NIH made in FY 2008. Candidate climate and health projects were identified by an NIH grant-reporting tool, the NIH’s Research, Condition, and Disease Categorization (RCDC) system (NIH 2012d; described in NIH 2010a). The NIH RCDC system automates and standardizes the NIH’s classification of grants into specific research areas, conditions, and diseases. Using its text mining software, the RCDC system compares “fingerprints” of weighted “concepts” (words or phrases) associated with research areas, conditions, or diseases with a grant’s concepts (based on the grant’s title, abstract, and specific aims) and decides whether or not that grant belongs to the category associated with that fingerprint.

In response to requests for information about federal expenditures in climate change over the past 20 years from Congress, the Executive Office of the President, and the public, three RCDC categories related to climate were developed. These categories are Climate Change, Global Warming Climate Change, and Climate-Related Exposures and Conditions (this last category was renamed as of FY 2012 from its earlier title “Health Effects of Climate Change” to more accurately reflect the content of the research conducted—the earlier title will be used because this document refers to FY 2008 data). Climate Change and Global Warming Climate Change were defined relatively narrowly, in response to the requests for such categories. The category with the largest number and dollar value of funded projects, Health Effects of Climate Change, was defined to address more broadly the health changes that are likely to occur as a result of climate change, and what research is being done on those health issues. RCDC fingerprints were developed for each of these categories when the NIH adopted the RCDC technology for portfolio reporting.

For the analysis described here, we developed a new fingerprint that combined these three fingerprints. The largest weight associated with a concept that appeared in more than one of these fingerprints was assigned to that concept in the new fingerprint. In addition, because the historical fingerprints, even when combined, might not capture the entire spectrum of current climate change–relevant research, additional concepts were added as maximally weighted Boolean terms based on an analysis of the National Library of Medicine (NLM) medical subject headings (MeSH) terms (NLM’s controlled vocabulary thesaurus; NLM 2009) associated with references cited in the health chapter of the 2008 CCSP report *Analyses of the Effects of Global Change on Human Health and Welfare and Human Systems* (Ebi et al. 2008). Applying this new fingerprint to the list of projects funded in FY 2008 tracked by the NIH RCDC returned 1,269 projects; few were captured as a result of the addition of the Boolean terms.

To ensure that the list of grants to be analyzed included all relevant projects, representatives from the ICs that funded projects in the list evaluated their IC’s projects and added 86 projects not captured by the new fingerprint but deemed relevant, resulting in a total of 1,357 projects. For multicomponent grants, subprojects were captured and categorized. Because the projects captured through this approach were individual awards, including supplemental awards to existing grants, there was some duplication of project titles, but not of funded activities.

Given the broad and historical concepts in the fingerprint used in compiling the initial project list, not all projects were equally relevant to current research questions on climate change and health. To assess the relevance of each project to climate change and health, we defined four “tiers,” ranging from tier 1 for most relevant to tier 4 for not relevant to climate and human health. These definitions allowed us to differentiate among research projects on health directly related to climate projections or climate variability (tier 1), research projects that examined the effects of climate variables [e.g., rainfall, temperature, humidity, ultraviolet (UV) radiation exposure] on health or disease without directly addressing the influence of climate change or climate variability (tier 2), and research projects on health outcomes for which climate is likely to be an important driver, but in which climate variables were not explicitly addressed, measured, or estimated (tier 3). Tier 4 comprised projects that were not relevant to climate change and health. IC representatives reviewed each of their respective projects and assigned a tier based on the project’s abstract and specific aims, and their knowledge of the project itself. We reviewed these project tier assignments, and in a few cases made adjustments, in consultation with IC representatives. Two of the 86 added projects were classified as tier 1, 5 as tier 2, and 79 as tier 3. In the total project list (1,357 projects), 7 projects were classified as tier 1, 85 as tier 2, and 706 as tier 3 (Table 1); 559 projects were classified as tier 4 (not relevant to climate change and health). A list of tier 1 and tier 2 projects is provided in Supplemental Material, Tables S1 and S2 (http://dx.doi.org/10.1289/ehp.1104518).

**Table 1:** Distribution of climate change and human health projects across the NIH funded from FY 2008 appropriations according to their relevance (n).

NIH institute, center, or office	Tier 1	Tier 2	Tier 3
Fogarty International Center (FIC)	2	6	30
National Cancer Institute (NCI)		9	140
National Center for Complementary and Alternative Medicine (NCCAM)			8
National Center for Research Resources (NCRR)		2	41
National Center on Minority Health and Health Disparities (NCMHD)		1	5
National Eye Institute (NEI)		5	1
National Heart, Lung, and Blood Institute (NHLBI)		3	8
National Human Genome Research Institute (NHGRI)			3
National Institute of Allergy and Infectious Diseases (NIAID)	3	9	28
National Institute of Arthritis and Musculoskeletal and Skin Diseases (NIAMS)		12	9
National Institute of Biomedical Imaging and Bioengineering (NIBIB)			1
National Institute of Child Health and Human Development (NICHD)		5	60
National Institute of Dental and Craniofacial Research (NIDCR)
National Institute of Diabetes and Digestive and Kidney Diseases (NIDDK)			4
National Institute of Environmental Health Science (NIEHS)		22	327
National Institute of General Medical Sciences (NIGMS)	2	1	16
National Institute of Mental Health (NIMH)		3	3
National Institute of Neurological Disorders and Stroke (NINDS)			3
National Institute of Nursing Research (NINR)			3
National Institute on Aging (NIA)		3	7
National Institute on Alcohol Abuse and Alcoholism (NIAAA)
National Institute on Deafness and Other Communication Disorders (NIDCD)
National Institute on Drug Abuse (NIDA)		4
National Library of Medicine (NLM)			5
Office of the Director, NIH (OD)			4
Tier 1 includes studies on health impact or interventions directly related to climate projections and studies of the impacts of interannual variability in environmental factors; tier 2 includes studies on the effects of climate variables (e.g., temperature, UV, humidity, rainfall, precipitation, weather, carbon dioxide) on biological systems, disease, and public health; and tier 3 includes studies of disease, biological systems, and public health needs very likely to be affected by climate, but climate variables are not explicitly addressed, measured, or estimated in the project.			

We reviewed the abstracts and specific aims of the 92 projects in tiers 1 and 2 to further categorize the types of projects by climate pathway, health impact, population, study type, and objective. The most relevant category or, in many cases, categories were assigned to each project. For example, a project might be identified as having both laboratory and mathematical modeling study types.

## Results

Although many NIH-funded projects were indirectly related to climate change, a much smaller number of projects explicitly referred to climate change or attempted to project its effects. The seven tier 1 (climate change–focused) projects and the 85 tier 2 (climate change–related) projects represented awards from 14 NIH ICs. Among all three tiers, a total of 21 ICs and the Office of the Director were represented (Table 1). The seven tier 1 projects included a study of the ecology of schistosomiasis in coastal Kenya in relation to climate variability (funded by the Fogarty International Center), a study of the ecology of African highland malaria that examined climate change as one of several factors influencing malaria’s epidemiology [funded by the National Institute of Allergy and Infectious Diseases (NIAID)], the development of a web-based decision support system for disease outbreak forecasting that integrates remotely sensed data, including climate data, with other environmental and public health data (funded by NIAID), and the development of a predictive model to describe the effects of climate change and other factors on the distribution of tsetse flies and African trypanosomiasis (sleeping sickness) across Kenya (funded by the National Institute of General Medical Sciences).

**Figure 1:**
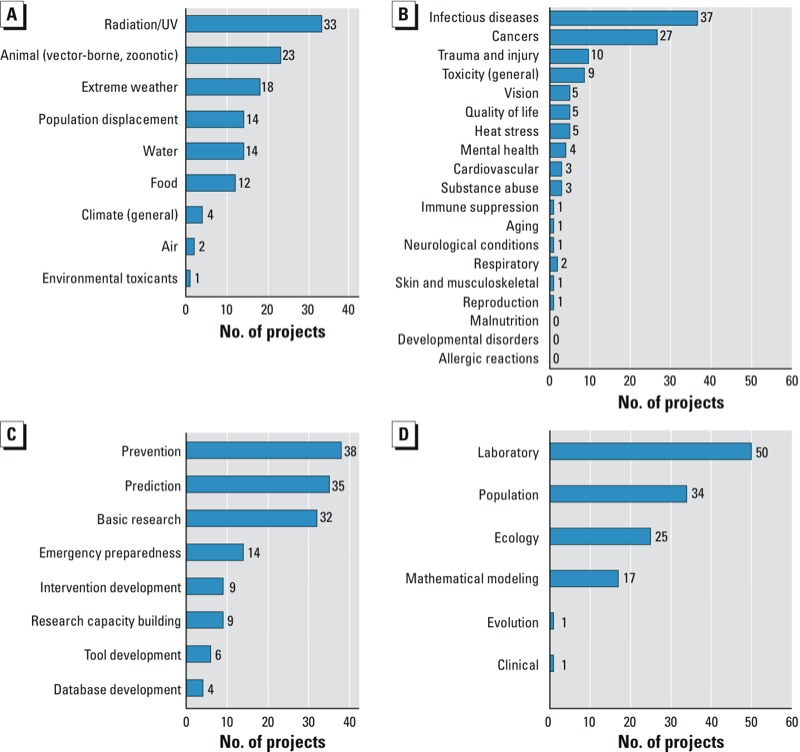
Classification of tier 1 and tier 2 projects (total) according to (*A*) exposure pathway, (*B*) health impact, (*C*) study objective, and (*D*) study type. Project categories are not mutually exclusive (e.g., a project that addresses multiple categories is counted in each relevant category).

Considering tiers 1 and 2, the NIH-funded projects examined many different health impacts (infectious diseases, trauma/injury, cancers) in relation to a variety of exposure pathways (extreme weather, vector-borne and zoonotic diseases, UV radiation), using a variety of study types and objectives (Figure 1). Most projects in tiers 1 and 2 addressed cancers (radiation/UV) or infectious diseases (particularly vector-borne or zoonotic), but other health impacts were also represented. The most common study objectives were prediction, prevention, and basic research, but some projects focused on emergency preparedness. Some projects included the development of forecasting tools that used environmental data to predict pathogen outbreaks, some focused on emergency preparedness for extreme weather events, and others involved developing databases to help improve the assessment of air pollution impacts on morbidity and mortality. The study types included laboratory experiments, population studies, field ecology, and mathematical modeling. Examples included laboratory studies of UV exposure, population studies that examined resilience after extreme weather events, ecological studies that examined the biology of pathogens and vectors in the field, and modeling efforts that focused on evaluating and forecasting disease risk. Of the nearly 53,000 awards that NIH made in 2008, tier 1 and 2 projects made up approximately 0.17% of the NIH portfolio.

The 706 climate-relevant projects in tier 3, which were broadly distributed across NIH ICs, focused on topics such as infectious diseases, respiratory diseases and asthma, and exposure to environmental toxins. Although these projects did not explicitly address climate change, the RFAs and PAs under which these studies were solicited contribute to a knowledge base about climate-related health conditions and further the identification of a community of scientists whose work could expand to include climate effects.

Some features of the tiers 1, 2, and 3 projects are noteworthy. Of those tier 1 and tier 2 projects that explicitly addressed specific populations, several focused on groups identified as being especially vulnerable to the impacts of climate change, such as impoverished populations, children, and seniors. Similarly, although many studies focused on populations in the United States, with many related to the aftermath of Hurricane Katrina, or were based in U.S. laboratories, several studies had an international focus or component, including developing countries and Arctic regions, which are considered most vulnerable to climate change (Anisimov et al. 2007; United Nations Framework Convention on Climate Change 2007). Of the 92 tier 1 and 2 projects, 30 included a domestic focus, and 21 projects included an international focus; the other projects did not have an explicit geographic focus. Finally, although most projects addressed impacts and adaptation, some addressed climate change mitigation, such as a study of basic microbial metabolism related to alternative energy production.

## Discussion

As climate change is increasingly recognized as a global problem, better understanding of its potential impacts on human health gains urgency. Improved scientific understanding of the relationship between climate change and health can contribute to developing interventions to reduce vulnerability to climate change and ensuring that mitigation efforts to reduce greenhouse gas emissions address health impacts.

The results of this analysis suggest some relatively straightforward, and in some cases low-cost, approaches to advancing climate and health research at the NIH. For example, through identifying individual grants on the human health effects of climate change, this analysis enabled us to also identify the funding programs that have led to relevant and successful grant applications. By highlighting these relevant opportunities [e.g., in a Notice in the NIH *Guide for Grants and Contracts* (NIH 2010c)], we have sought to encourage greater use of existing funding opportunities to expand the NIH portfolio in this area.

In addition, the analysis revealed that the three RCDC categories that the agency uses to identify and report climate and health grants could possibly be refined: 86 projects identified by IC representatives were not captured in the combined category, and > 40% of the projects captured in the combined category were deemed not to be relevant to climate and health upon manual examination (559 tier 4 projects). Whether research on UV radiation exposure related to stratospheric ozone depletion is relevant to climate change is controversial. Because the USGCRP considers both climate change and stratospheric ozone depletion to be aspects of global change, NIH has traditionally included a substantial portion of its UV effects studies in its climate change portfolio, which is reported to the USGCRP. Although accumulating greenhouse gases also contribute to stratospheric ozone depletion by enhancing the catalyzed destruction of ozone by chlorofluorocarbons, UV-induced skin cancer is not generally considered a consequence of global climate change. In keeping with historic NIH reporting precedence, however, we have included skin cancer studies in our analysis, but have excluded studies in which UV radiation was used solely as a DNA damaging agent in laboratory settings to understand basic cellular mechanisms. Were studies of ultraviolet exposure and associated skin cancers to be omitted from our lists, the number of overall studies specific to global climate change health effects would be smaller. Specifically, the total number of tier 1 and tier 2 projects would be 59 instead of 92. The distribution of projects into subcategories would also change: The number of cancer-related projects would drop to nearly zero, the number of laboratory studies would be reduced by half, and the representation of basic research would be reduced by two-thirds.

Research funding on climate change and health in countries other than the United States appears to be increasing. Although data are not readily available for all countries that note ongoing research on the topic, a recent review from Canada (Ford et al. 2011) and the Medical Research Council of Australia’s website (National Health and Medical Research Council 2011) demonstrate significant growth over the past decade. Canada’s investment rose from $160,000 (Canadian dollars) for five projects in 1999–2000 to around $5 million in 2008–2009, supporting 45 projects. Similarly, Australia’s research funding grew from $124,000 supporting one project in 2002 to $1.2 million (Australian dollars) supporting seven projects in 2008, and a forecast of $2.6 million for 14 projects in 2011.

The growing interest in addressing climate and health research needs has led to increased activities at the NIH. Through its basic and clinical research mission and portfolio, the NIH has been increasing its investment in research to understand how the complex interrelationships among humans, ecosystems, climate, climate variability, and climate change are affecting and may continue to affect both domestic and global health. The NIH has begun to invest explicitly in priority climate and health research through targeted funding opportunities.

The Trans-NIH Working Group on Climate Change and Health (NIH 2012e) was formed in 2007, and has facilitated the development of several recent climate-related funding announcements. In 2009, the NIH released an announcement to fund climate change and health research projects through the American Recovery and Reinvestment Act (ARRA). Five projects were funded through this solicitation (see ARRA 2010 for a description of the activities). These projects focused on how current climate and future climate change might affect human health, including heat-related morbidity and mortality, respiratory and cardiovascular impacts of air pollution (especially during wildfires), cholera, and mass human migration.

In June 2010, the NIH released a *Notice to Highlight Current NIH Funding Opportunities that Promote Research on the Human Health Effects of Climate Change* (NOT-TW-10-008; NIH 2010b). The notice identified five funding opportunity announcements linked to previous support for climate change and health research, and seven indirectly related to climate change and health research.

In July 2010, the NIH’s National Institute of Environmental Health Sciences (NIEHS) released a funding opportunity announcement titled *Climate Change and Health: Assessing and Modeling Population Vulnerability to Climate Change* (NIH 2010c), in which eight other NIH ICs participated. The announcement encouraged the submission of multidisciplinary research proposals to examine the differential risk factors of populations that are associated with increased vulnerability to climate change in order to help inform climate change adaptation and public health interventions to reduce current and future vulnerability of various populations to the health effects of climate change.

Although interest in the health impacts of climate change is growing, and funding for research is gradually increasing, the broad, complex, and mostly indirect relationship between climate and human health, and the multidecadal time scale of climate change pose challenges for biomedical research. Furthermore, the overwhelming majority of NIH research is on basic biology underlying health and disease. Population-based epidemiology and intervention studies of the sort that are most closely aligned to the questions of climate change impact and adaptation needs have historically been a modest portion of the NIH portfolio. For example, another population-based field, rural health, funded approximately 481 grants in the same year as those in our study (NIH 2012b), which is more than the 85 projects in our tiers 1 and 2, but less than the 798 projects in tiers 1, 2, and 3 in our study. However, as a result of internal discussions as well as calls from the general public, Congress, and the research community, NIH has in recent years increasingly invested in translational research (e.g., NIH 2011) and implementation science (Madon et al. 2007).

The topic of climate change and health, whose burdens are diffuse and largely projected, is in many ways more challenging to address than emerging biomedical issues whose disease burdens are more immediate and apparent. Research on climate and health requires multidisciplinary teams that can include meteorologists, climatologists, mathematicians and statisticians, computational scientists, and a diversity of health scientists. Throughout research there is a growing emphasis on team science and the recognition of its importance to productivity and its role in solving large problems (e.g., Hall et al. 2012). Nonetheless, a key challenge to climate and health research is that most researchers are not cross-trained in both the earth and health sciences, and they tend to ask different questions and work at different temporal and spatial scales. Therefore, there is a need to train individuals across disciplines and to fund multidisciplinary teams to help ensure effective collaboration and high-quality science. Agencies that support climate and health research might encourage such multidisciplinary research in their solicitations and peer review, and might collaborate more with agencies having complementary missions and expertise in climate science, mathematical modeling of populations, human health surveillance, and ecological and agricultural research.

Analyses such as the one presented here are part of the process of identifying and addressing research gaps. As is the case in the development of any new field, working with a broad research community to identify priority research needs and opportunities is required. To this end, in December 2009, the Trans-NIH Working Group on Climate Change and Health convened a group of health researchers and climate experts to present and discuss these needs and opportunities. Their Priorities for NIH Research on Climate Change and Health Workshop featured analyses of historical NIH activities in the area, and included presentations by recent NIH climate change and health grantees supported by ARRA challenge grant funding and other scientists from the extramural community (Taylor 2010). The workshop participants identified some priority research opportunities at the population level that informed the subsequent funding opportunity announcement (NIH 2010c), and these have continued to inform the development of climate change and health research activities at the NIH. We have also opened a discussion with science and policy communities about government-wide needs and opportunities and how the NIH’s particular strengths in human health research can best contribute to understanding the implications of global climate change.

We conclude with some observations. First, similar to other areas of biomedical sciences, fiscal resources for making desirable investments to advance this field are likely to be constrained in the near to medium term by broad fiscal pressures on all government funding agencies. Second, many NIH ICs have roles in this field because of the diversity of potential health effects and their associated research needs. Third, the complexity of the field and the need for multidisciplinary collaboration with scientists beyond the traditional NIH-supported community calls for improved coordination to encourage the participation of multiple relevant agencies in supporting climate and health research. Given the nature and scale of the potential effects of climate change on the planet and on human health, and the degree of uncertainty that we face about these effects, we think that these issues are worth addressing.

## Supplemental Material

(106 KB) PDF
