# Supplementary material for: Climate Change, Human Health, and Biomedical Research: Analysis of the National Institutes of Health Research Portfolio
Source: Environ Health Perspect. 2013 Jan 18;121(4):399–404. doi: 10.1289/ehp.1104518 (PMC3620768; doi:10.1289/ehp.1104518)
Supplement: (106 KB) PDF [file ehp.1104518.s001.pdf]

Supplemental Material

Climate Change, Human Health, and Biomedical Research: Analysis of the National Institutes of Health Research Portfolio

Christine M. Jessup[1], John Balbus[2], Carole Christian[3], Ehsanul Haque[3], Sally E. Howe[4], Sheila A. Newton[2], Britt C. Reid[5], Luci Roberts[6], Erin Wilhelm[1], and Joshua P. Rosenthal[1]

[1] Fogarty International Center, National Institutes of Health, Bethesda, Maryland, USA; [2] National Institute of Environmental Health Sciences, National Institutes of Health, Bethesda, Maryland, USA; [3] Division of Program Coordination, Planning, and Strategic Initiatives, National Institutes of Health, Bethesda, Maryland, USA; [4] National Library of Medicine, National Institutes of Health, 8600 Rockville Pike, Bethesda, Maryland, USA; [5] National Cancer Institute, National Institutes of Health, Bethesda, Maryland, USA; [6] Office of Extramural Research, National Institutes of Health, Bethesda, Maryland, USA.

Table of Contents

Table S1. List of Tier 1 projects identified in the analysis of the FY 2008 project records stored in the NIH RCDC system database. 2

Table S2. List of Tier 2 projects identified in the analysis of the FY 2008 project records stored in the NIH RCDC system database. 3

Table S1. List of Tier 1 projects[a] identified in the analysis of the FY 2008 project records stored in the NIH RCDC system database.

Project	Title
1 R01AI079411-01	An Integrated System for the Epidemiological Application of Earth Observation Technologies
5 R21GM084704-02	Dynamic Ecological Simulation Model of Tsetse Transmitted Trypanosomosis in Kenya
3 R21GM084704-01S1	Dynamic Ecological Simulation Model of Tsetse Transmitted Trypanosomosis in Kenya
1R01TW008067-01	Eco-epidemiology of Schistosomiasis, Malaria and Polyparasitism in Coastal Kenya
2 R01AI050243-07A2	Ecology of African Highland Malaria
3 R01AI050243-07A2S1	Ecology of African Highland Malaria
5-D43-TW005750-07	UAB- International Training in Environmental and Occupational Health (ITREOH) Program

[a] Tier 1 projects include studies on health impact or interventions directly related to climate projections and studies of the impacts of inter-annual variability in environmental factors. Note that the same project may appear multiple times because project supplements were awarded during the time period being analyzed. The duplication of project titles does not reflect duplication of funded activities.

Table S2. List of Tier 2 projects[a] identified in the analysis of the FY 2008 project records stored in the NIH RCDC system database.

Project	Title
5 R01AG024373-05	Adaptive Physiology of mtDNA Longevity Mutations
1 R01HD057599-01	Adversity and Resilience after Hurricane Katrina
5 R01AG028621-02	Age, Disruption, and Life Reorganization after Hurricane Katrina
5 P50ES012742-05	Alexandrium Population Biology in the Gulf of Maine
5 R01ES015375-04	Apple Peel Extract Protects UV-Induced Carcinogenesis
5 R01AR054856-02	Calcineurin signaling in the keratinocyte UVB response
5 P01ES014635-02	Cell Cycle Check Points
5 P50ES012762-05	Core--Environmental Facility
5 P50ES012736-05	Core--Remote Sensing
5R01EY013127-08	Corneal-Epithelial Nuclear Ferritin and U.V. Protection
1 R01ES015552-01A1	Defining critical aspects of environmental ultraviolet exposure in melanogenesis
5 R01ES013679-03	Developing genomic resources for the toxic dinoflagellate Alexandrium tamarense
5 R01ES012459-05	Domoic Acid Neurotoxicity in Native Americans
3 R01ES012459-05S1	Domoic Acid Neurotoxicity in Native Americans
5 R01MH078152-03	Ecological Approaches to Understanding Post-Disaster Distress
5 R01AI049724-08	Ecology of Encephalitis Viruses in the USA
5 R01TW007869-03	Ecology-Based Risk Assessment and Early Warning for HPAI in Asia
3 R01TW007869-03S1	Ecology-Based Risk Assessment and Early Warning for HPAI in Asia
3 R01TW007869-03S2	Ecology-Based Risk Assessment and Early Warning for HPAI in Asia

Project	Title
5R01EY018100-02	Elevated potassium ion in lacrimal fluid and the health of the ocular surface
5 R21DA023045-02	Emergency management for disruptions in methadone treatment.
5 P30AR039750-18	Epigenetic Responses to Solar UVR in Melanocytes
5 R01DA021887-03	Family-Based Drug Services for Young Disaster Victims
5 K01ES014003-04	Gene-environment Cancer Risks in Melanoma
2 R01HL061388-09	Heat Stress and Circulatory Control
5 P50ES012742-05	Human Pathogens and Coastal Ocean Processes
1 R01MH081832-01	Hurricane Katrina Community Advisory Group
1 P30MH082760-01	Hurricane Katrina Community Analysis Group Pilot
Y1MD5024-9-0-1	Hurricane Katrina Relief Efforts
3 R01TW006986-04S1	Impact of Land Cover Change on Hantavirus Ecology
5 R01CA106807-05	Indoor Tanning Use, DNA Repair and Risk of Melanoma
1 Z01AI000996-02	Influenza Viral Genomics and Evolution
5 R01AI044793-08	Invasion Biology of Aedes albopictus
5 R01HL065599-08	Mechanisms of Cutaneous Active Vasodilation
5R01EY018343-02	Mechanisms of Environmental Stress Affecting Corneal Epithelial Wound Healing
1 R01ES015585-01A1	Mechanisms of UV-induced skin carcinogenesis
5 P50ES012740-05	Microbial Pathogens in Tropical Coastal Waters
5 P50ES012742-05	Microecology and Evolution of Two Marine Pathogens
5 R01AR043369-12	Mitf: A Master Gene for Melanocyte Development

Project	Title
5 R01AR051552-03	Mitochondrial DNA Mutations in Keratinocyte Hyperplasia
5 R01AI042164-10	Modeling and empirical studies of arboviruses in Florida
1 R01GM085335-01A2	Molecular genetics of thermotaxis
1 R21AI078463-01A1	Molecular Markers for Anopheles Gambiae Age Grading
5 R01AI058279-04	Molecular regulation of diapause in Culex pipiens
5 R01EY011490-10	Molecular Mechanisms & Role of Corneal Aldehyde Dehydrogenase
5 P01ES014635-02	Murine & Human In Vivo Models of Melanoma Formation
5 R01ES015028-03	National Assessment of the Mortality and Morbidity Effects of Tropospheric Ozone
5 R01HL084072-03	Neural and non-neural modulators of skin blood flow and sweating in humans
5 R01CA057494-14	Non-Melanoma Skin Cancer in New Hampshire
5 R01AR052190-03	Novel Biosynthetic Pathway for Secosteroids in the Skin
5 P50ES012740-05	Pacific Research Center for Marine Biomedicine - Research Project 1: Ciguatera-Dinoflagellate Nutrient Profile and Ecology
5 D43TW001505-09	Population Biology of African Malaria Vectors
1 K01HD055415-01A1	Population-environment dynamics influencing malaria risk in the Peruvian Amazon
1 Z01AI000208-28	Protein Synthesis And Developmental Transitions In Plasmodium
5 R01CA101602-05	Quantification of the environmental UVA exposure
5 K25AR050397-04	Quantitative Characterization of UV induced skin damage
5 P41RR002594-23	Raman spectroscopy studies of tropical, temperate and polar corals
5 P50ES012736-05	Recreational microbes: indicators for monitoring water quality
1 R01EY018177-01A1	Regulation of UV-induced apoptosis

Project	Title
1 R21HD059087-01	Resettlement and Well-Being of New Orleans Residents After Hurricane Katrina
5 P60AR047785-08	Risk Factors for Gout Attacks: A Case-crossover Study
5 K08AI067549-02	Risk for Future Outbreaks of Henipaviruses in South Asia
5-R03-AR-53710-02	Role of PPARgamma in ultraviolet stress responses
5 P20RR016466-08	S. parryii – A model hibernator physiology
1 R01AG030619-01A2	SAFEHAVEN: Decision Support for Nursing Home Resident Disaster Evacuations
5 P50ES012762-05	Shellfish Kinetics
2 R01ES009110-10	Signaling Pathways for UV-Induced Melanogenic Response
1 R21AR054361-01A2	Slug Mediates the Acute Cutaneous Response to UVR
5 R01CA112524-04	Solar Exposure and Melanoma Survival
5 K25AI067791-03	Spatially-explicit mathematical model of human monocytic ehrlichiosis
1 Z01CP010135-13	Studies of Non-Ionizing Radiation-Related Cancer
1 Z01CP010132-13	Studies of Populations Exposed to Environmental Sources of Radiation
5 R01TW007248-02	Study of risk factors of tick-borne encephalitis in Poland
5 R01DA021852-03	Substance Use and Other Health Consequences Among Katrina Evacuees in Houston
3 R01DA021852-03S1	Substance Use and Other Health Consequences Among Katrina Evacuees in Houston
5 K07CA104556-04	Sun Exposure and Melanoma in Agricultural Workers
1 R01HD059106-01	Survey of New Orleans Residents Displaced by Hurricane Katrina
1 R21HD057608-01	The Displaced New Orleans Residents Pilot Study
2 R56AR049342-06	The Framingham School Study of Nevi in Children: Sonic II

Project	Title
5 P30AR050948-05	The Innate Immune System in Regulation of DVB Induced Skin Carcinogenesis
5-K02-AR-50993-06	The replication checkpoint and genomic fidelity in skin
1 R21ES015832-01A2	The role of E-cadherin in photocarcinogenesis
5 P50ES012736-05	Toxic Harmful Algal Blooms (HABs)
5 R01CA077646-09	Ultraviolet-Induced Signal Transduction
2 R01CA086928-06A2	UV-induced and NOS-mediated Zn elevation, translation regulation and apoptosis

[a] Tier 2 projects include studies on the effects of climate variables (e.g., temperature, UV, humidity, rainfall, precipitation, weather, CO2) on biological systems, disease, and public health. Note that the same project may appear multiple times because project supplements were awarded during the time period being analyzed. The duplication of project titles does not reflect duplication of funded activities.
